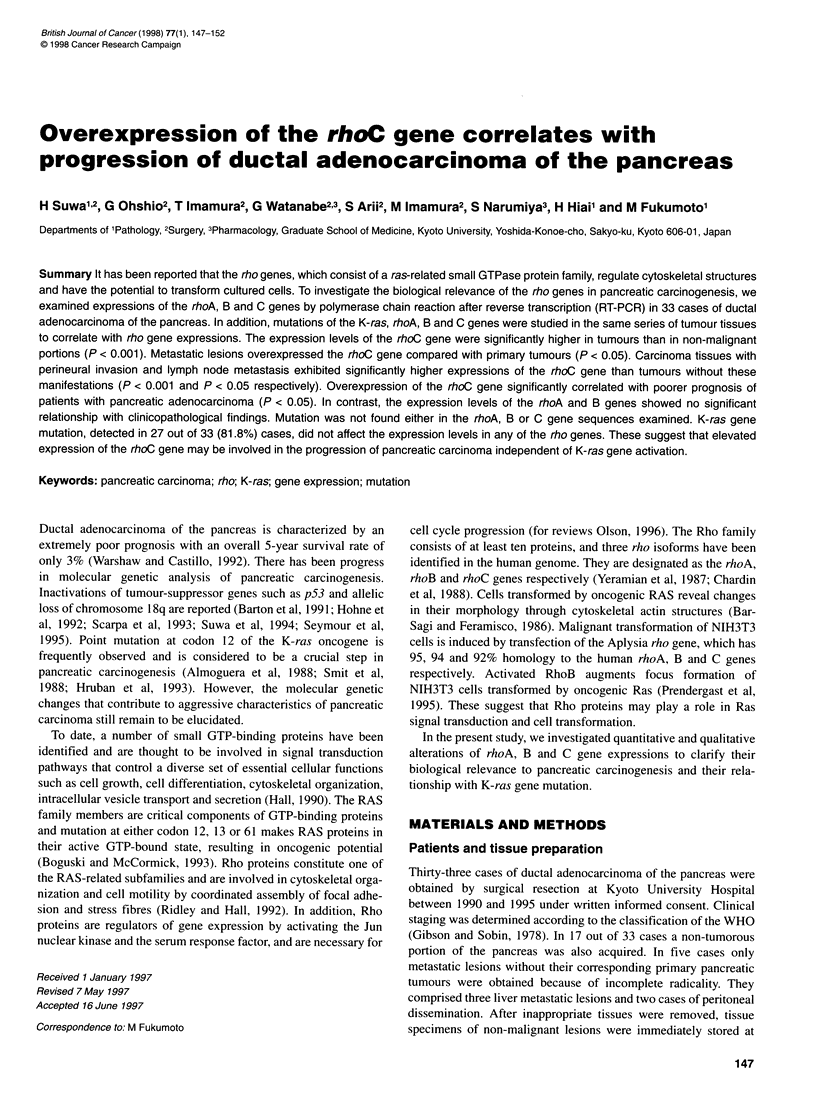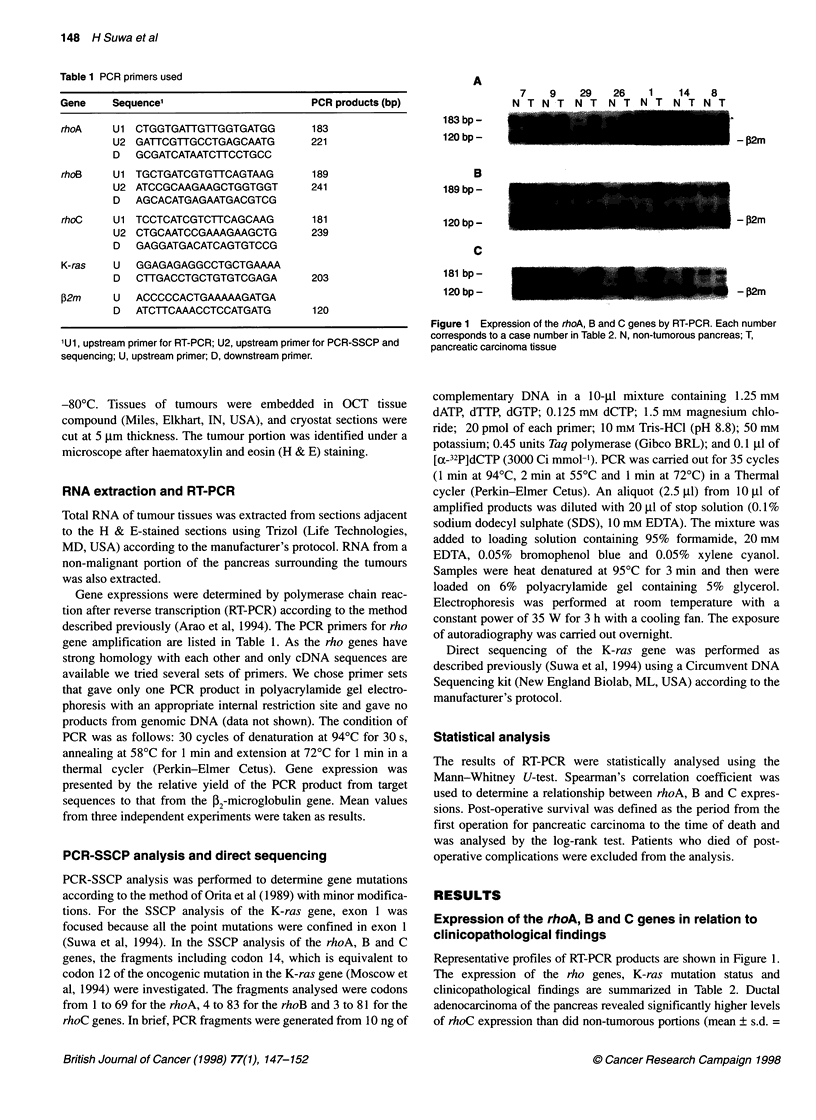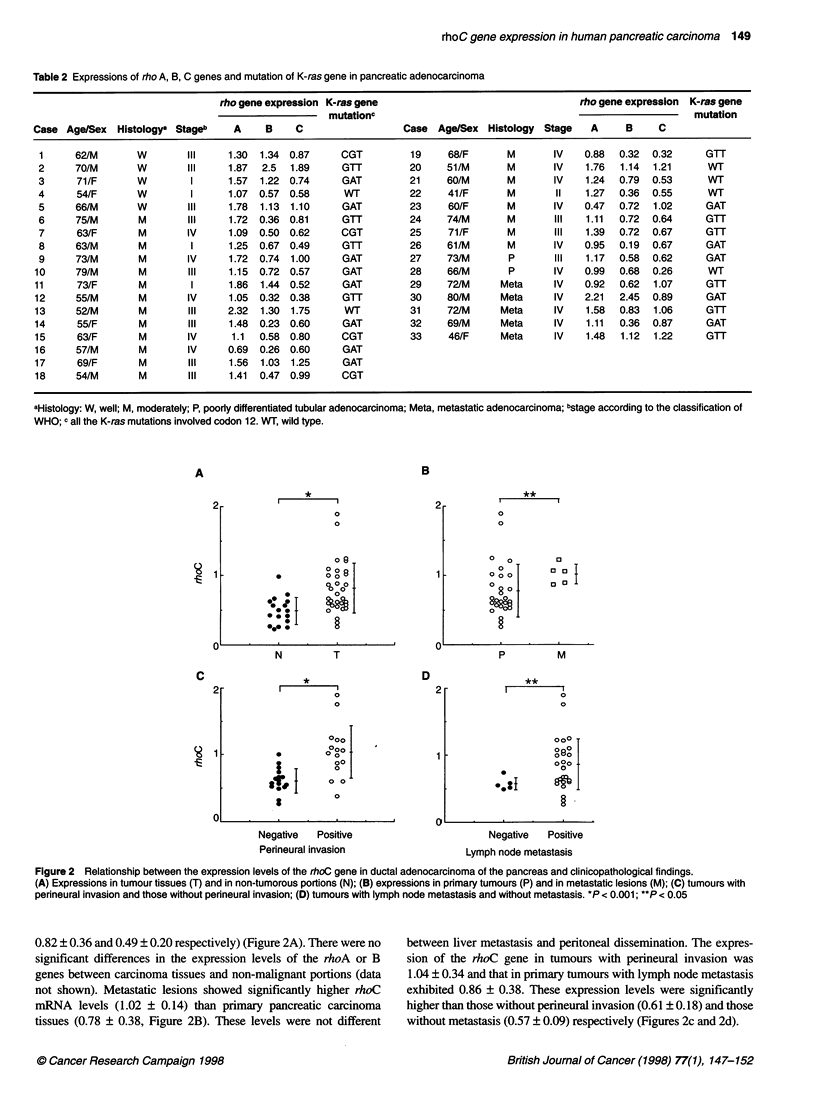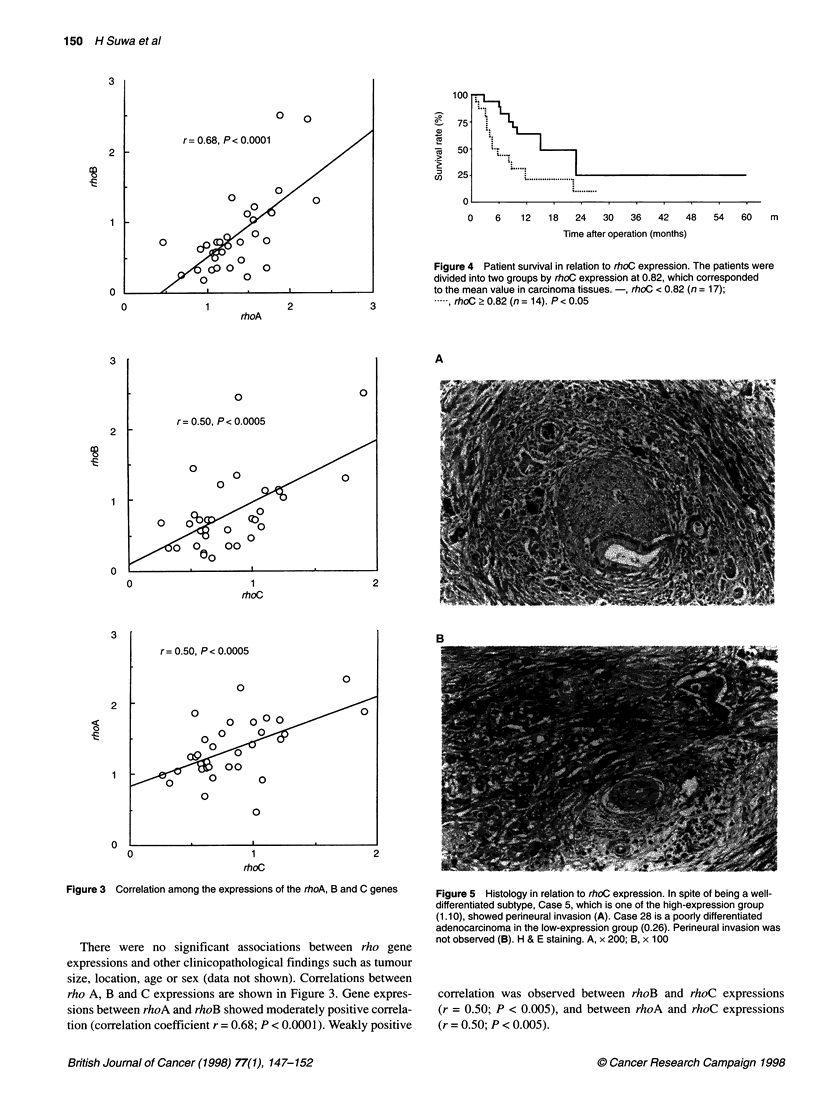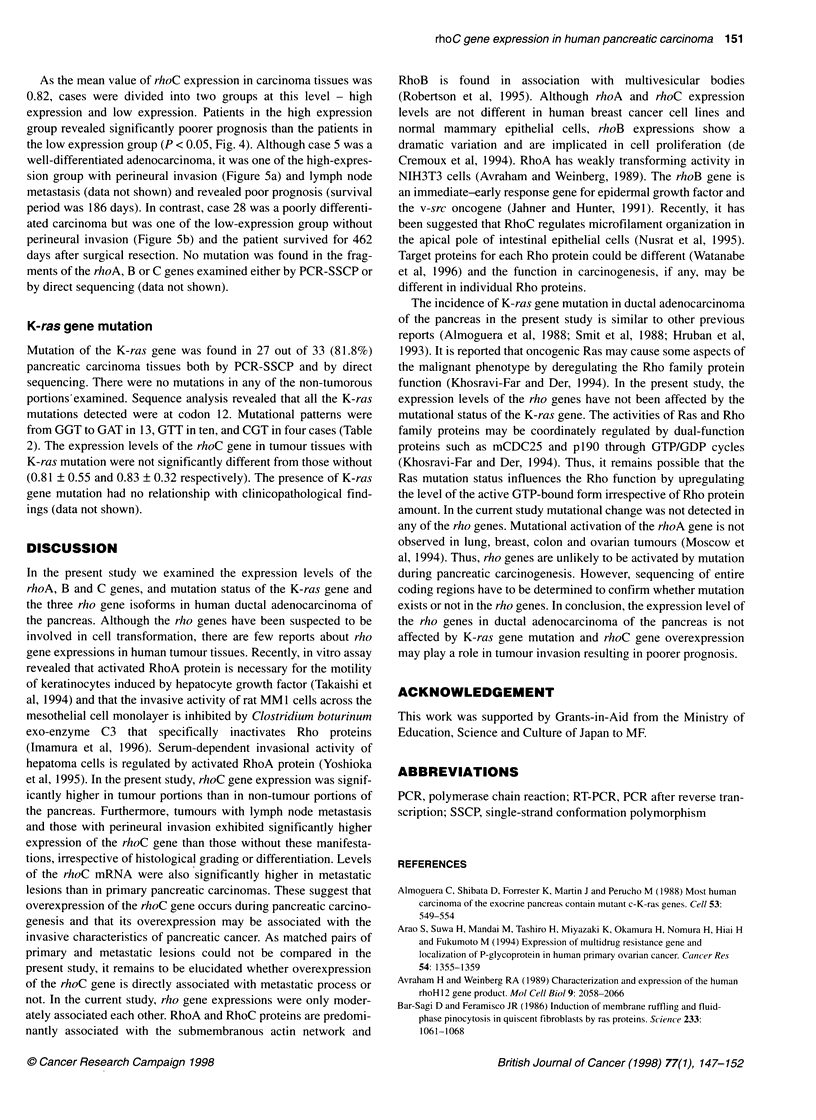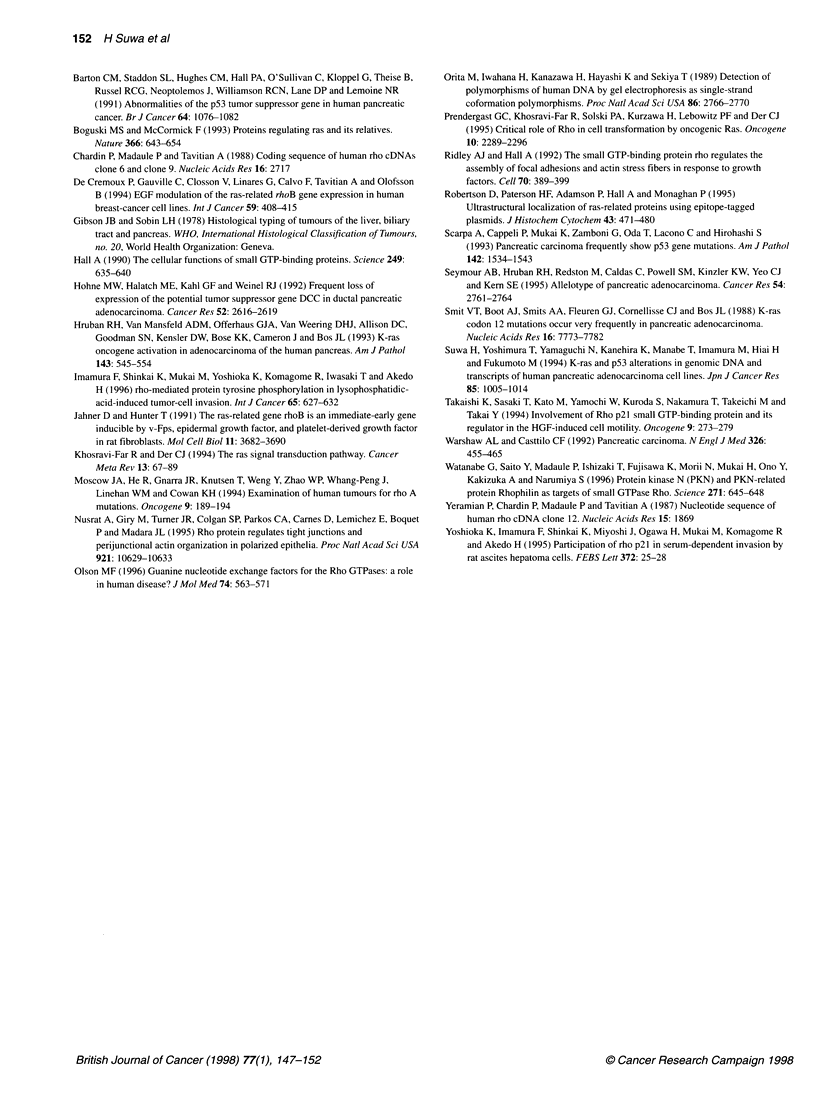# Overexpression of the rhoC gene correlates with progression of ductal adenocarcinoma of the pancreas.

**DOI:** 10.1038/bjc.1998.23

**Published:** 1998

**Authors:** H. Suwa, G. Ohshio, T. Imamura, G. Watanabe, S. Arii, M. Imamura, S. Narumiya, H. Hiai, M. Fukumoto

**Affiliations:** Department of Pathology, Graduate School of Medicine, Kyoto University, Japan.

## Abstract

**Images:**


British Journal of Cancer (1998) 77(1), 147–152
© 1998 Cancer Research Campaign

### Overexpression of the *rhoC* gene correlates with progression of ductal adenocarcinoma of the pancreas

**H Suwa[1,2], G Ohshio[2], T Imamura[2], G Watanabe[2,3], S Arii[2], M Imamura[2], S Narumiya[3], H Hiai[1] and M Fukumoto[1]**

Departments of [1]Pathology, [2]Surgery, [3]Pharmacology, Graduate School of Medicine, Kyoto University, Yoshida-Konoe-cho, Sakyo-ku, Kyoto 606-01, Japan

**Summary** It has been reported that the *rho* genes, which consist of a *ras*-related small GTPase protein family, regulate cytoskeletal structures and have the potential to transform cultured cells. To investigate the biological relevance of the *rho* genes in pancreatic carcinogenesis, we examined expressions of the *rho*A, B and C genes by polymerase chain reaction after reverse transcription (RT-PCR) in 33 cases of ductal adenocarcinoma of the pancreas. In addition, mutations of the K-*ras*, *rho*A, B and C genes were studied in the same series of tumour tissues to correlate with *rho* gene expressions. The expression levels of the *rho*C gene were significantly higher in tumours than in non-malignant portions ($P < 0.001$). Metastatic lesions overexpressed the *rho*C gene compared with primary tumours ($P < 0.05$). Carcinoma tissues with perineural invasion and lymph node metastasis exhibited significantly higher expressions of the *rho*C gene than tumours without these manifestations ($P < 0.001$ and $P < 0.05$ respectively). Overexpression of the *rho*C gene significantly correlated with poorer prognosis of patients with pancreatic adenocarcinoma ($P < 0.05$). In contrast, the expression levels of the *rho*A and B genes showed no significant relationship with clinicopathological findings. Mutation was not found either in the *rho*A, B or C gene sequences examined. K-*ras* gene mutation, detected in 27 out of 33 (81.8%) cases, did not affect the expression levels in any of the *rho* genes. These suggest that elevated expression of the *rho*C gene may be involved in the progression of pancreatic carcinoma independent of K-*ras* gene activation.

**Keywords:** pancreatic carcinoma; *rho*; K-*ras*; gene expression; mutation

Ductal adenocarcinoma of the pancreas is characterized by an extremely poor prognosis with an overall 5-year survival rate of only 3% (Warshaw and Castillo, 1992). There has been progress in molecular genetic analysis of pancreatic carcinogenesis. Inactivations of tumour-suppressor genes such as *p53* and allelic loss of chromosome 18q are reported (Barton et al, 1991; Hohne et al, 1992; Scarpa et al, 1993; Suwa et al, 1994; Seymour et al, 1995). Point mutation at codon 12 of the K-*ras* oncogene is frequently observed and is considered to be a crucial step in pancreatic carcinogenesis (Almoguera et al, 1988; Smit et al, 1988; Hruban et al, 1993). However, the molecular genetic changes that contribute to aggressive characteristics of pancreatic carcinoma still remain to be elucidated.

To date, a number of small GTP-binding proteins have been identified and are thought to be involved in signal transduction pathways that control a diverse set of essential cellular functions such as cell growth, cell differentiation, cytoskeletal organization, intracellular vesicle transport and secretion (Hall, 1990). The RAS family members are critical components of GTP-binding proteins and mutation at either codon 12, 13 or 61 makes RAS proteins in their active GTP-bound state, resulting in oncogenic potential (Boguski and McCormick, 1993). Rho proteins constitute one of the RAS-related subfamilies and are involved in cytoskeletal organization and cell motility by coordinated assembly of focal adhesion and stress fibres (Ridley and Hall, 1992). In addition, Rho proteins are regulators of gene expression by activating the Jun nuclear kinase and the serum response factor, and are necessary for cell cycle progression (for reviews Olson, 1996). The Rho family consists of at least ten proteins, and three *rho* isoforms have been identified in the human genome. They are designated as the *rho*A, *rho*B and *rho*C genes respectively (Yeramian et al, 1987; Chardin et al, 1988). Cells transformed by oncogenic RAS reveal changes in their morphology through cytoskeletal actin structures (Bar-Sagi and Feramisco, 1986). Malignant transformation of NIH3T3 cells is induced by transfection of the Aplysia *rho* gene, which has 95, 94 and 92% homology to the human *rho*A, B and C genes respectively. Activated RhoB augments focus formation of NIH3T3 cells transformed by oncogenic Ras (Prendergast et al, 1995). These suggest that Rho proteins may play a role in Ras signal transduction and cell transformation.

In the present study, we investigated quantitative and qualitative alterations of *rho*A, B and C gene expressions to clarify their biological relevance to pancreatic carcinogenesis and their relationship with K-*ras* gene mutation.

*Received 1 January 1997*
*Revised 7 May 1997*
*Accepted 16 June 1997*

*Correspondence to:* M Fukumoto

### MATERIALS AND METHODS

### Patients and tissue preparation

Thirty-three cases of ductal adenocarcinoma of the pancreas were obtained by surgical resection at Kyoto University Hospital between 1990 and 1995 under written informed consent. Clinical staging was determined according to the classification of the WHO (Gibson and Sobin, 1978). In 17 out of 33 cases a non-tumorous portion of the pancreas was also acquired. In five cases only metastatic lesions without their corresponding primary pancreatic tumours were obtained because of incomplete radicality. They comprised three liver metastatic lesions and two cases of peritoneal dissemination. After inappropriate tissues were removed, tissue specimens of non-malignant lesions were immediately stored at

**Table 1** PCR primers used

| Gene | Sequence[1] | PCR products (bp) |
|---|---|---|
| *rho*A | U1 CTGGTGATTGTTGGTGATGG | 183 |
| | U2 GATTCGTTGCCTGAGCAATG | 221 |
| | D GCGATCATAATCTTCCTGCC | |
| *rho*B | U1 TGCTGATCGTGTTCAGTAAG | 189 |
| | U2 ATCCGCAAGAAGCTGGTGGT | 241 |
| | D AGCACATGAGAATGACGTCG | |
| *rho*C | U1 TCCTCATCGTCTTCAGCAAG | 181 |
| | U2 CTGCAATCCGAAAGAAGCTG | 239 |
| | D GAGGATGACATCAGTGTCCG | |
| K-*ras* | U GGAGAGAGGCCTGCTGAAAA | |
| | D CTTGACCTGCTGTGTCGAGA | 203 |
| β2m | U ACCCCCACTGAAAAAGATGA | |
| | D ATCTTCAAACCTCCATGATG | 120 |

[1]U1, upstream primer for RT-PCR; U2, upstream primer for PCR-SSCP and sequencing; U, upstream primer; D, downstream primer.

**Figure 1** Expression of the *rho*A, B and C genes by RT-PCR. Each number corresponds to a case number in Table 2. N, non-tumorous pancreas; T, pancreatic carcinoma tissue

–80°C. Tissues of tumours were embedded in OCT tissue compound (Miles, Elkhart, IN, USA), and cryostat sections were cut at 5 μm thickness. The tumour portion was identified under a microscope after haematoxylin and eosin (H & E) staining.

### RNA extraction and RT-PCR

Total RNA of tumour tissues was extracted from sections adjacent to the H & E-stained sections using Trizol (Life Technologies, MD, USA) according to the manufacturer's protocol. RNA from a non-malignant portion of the pancreas surrounding the tumours was also extracted.

Gene expressions were determined by polymerase chain reaction after reverse transcription (RT-PCR) according to the method described previously (Arao et al, 1994). The PCR primers for *rho* gene amplification are listed in Table 1. As the *rho* genes have strong homology with each other and only cDNA sequences are available we tried several sets of primers. We chose primer sets that gave only one PCR product in polyacrylamide gel electrophoresis with an appropriate internal restriction site and gave no products from genomic DNA (data not shown). The condition of PCR was as follows: 30 cycles of denaturation at 94°C for 30 s, annealing at 58°C for 1 min and extension at 72°C for 1 min in a thermal cycler (Perkin–Elmer Cetus). Gene expression was presented by the relative yield of the PCR product from target sequences to that from the $\beta_2$-microglobulin gene. Mean values from three independent experiments were taken as results.

### PCR-SSCP analysis and direct sequencing

PCR-SSCP analysis was performed to determine gene mutations according to the method of Orita et al (1989) with minor modifications. For the SSCP analysis of the K-*ras* gene, exon 1 was focused because all the point mutations were confined in exon 1 (Suwa et al, 1994). In the SSCP analysis of the *rho*A, B and C genes, the fragments including codon 14, which is equivalent to codon 12 of the oncogenic mutation in the K-*ras* gene (Moscow et al, 1994) were investigated. The fragments analysed were codons from 1 to 69 for the *rho*A, 4 to 83 for the *rho*B and 3 to 81 for the *rho*C genes. In brief, PCR fragments were generated from 10 ng of complementary DNA in a 10-μl mixture containing 1.25 mM dATP, dTTP, dGTP; 0.125 mM dCTP; 1.5 mM magnesium chloride; 20 pmol of each primer; 10 mM Tris-HCl (pH 8.8); 50 mM potassium; 0.45 units *Taq* polymerase (Gibco BRL); and 0.1 μl of [$\alpha$-$^{32}$P]dCTP (3000 Ci $mmol^{-1}$). PCR was carried out for 35 cycles (1 min at 94°C, 2 min at 55°C and 1 min at 72°C) in a Thermal cycler (Perkin–Elmer Cetus). An aliquot (2.5 μl) from 10 μl of amplified products was diluted with 20 μl of stop solution (0.1% sodium dodecyl sulphate (SDS), 10 mM EDTA). The mixture was added to loading solution containing 95% formamide, 20 mM EDTA, 0.05% bromophenol blue and 0.05% xylene cyanol. Samples were heat denatured at 95°C for 3 min and then were loaded on 6% polyacrylamide gel containing 5% glycerol. Electrophoresis was performed at room temperature with a constant power of 35 W for 3 h with a cooling fan. The exposure of autoradiography was carried out overnight.

Direct sequencing of the K-*ras* gene was performed as described previously (Suwa et al, 1994) using a Circumvent DNA Sequencing kit (New England Biolab, ML, USA) according to the manufacturer's protocol.

### Statistical analysis

The results of RT-PCR were statistically analysed using the Mann–Whitney *U*-test. Spearman's correlation coefficient was used to determine a relationship between *rho*A, B and C expressions. Post-operative survival was defined as the period from the first operation for pancreatic carcinoma to the time of death and was analysed by the log-rank test. Patients who died of post-operative complications were excluded from the analysis.

### RESULTS

### Expression of the *rho*A, B and C genes in relation to clinicopathological findings

Representative profiles of RT-PCR products are shown in Figure 1. The expression of the *rho* genes, K-*ras* mutation status and clinicopathological findings are summarized in Table 2. Ductal adenocarcinoma of the pancreas revealed significantly higher levels of *rho*C expression than did non-tumorous portions (mean ± s.d. =

© Cancer Research Campaign 1998

**Table 2** Expressions of *rho* A, B, C genes and mutation of K-*ras* gene in pancreatic adenocarcinoma

| Case | Age/Sex | Histology[a] | Stage[b] | *rho* gene expression A | B | C | K-*ras* gene mutation[c] | Case | Age/Sex | Histology | Stage | *rho* gene expression A | B | C | K-*ras* gene mutation |
|---|---|---|---|---|---|---|---|---|---|---|---|---|---|---|---|
| 1 | 62/M | W | III | 1.30 | 1.34 | 0.87 | CGT | 19 | 68/F | M | IV | 0.88 | 0.32 | 0.32 | GTT |
| 2 | 70/M | W | III | 1.87 | 2.5 | 1.89 | GTT | 20 | 51/M | M | IV | 1.76 | 1.14 | 1.21 | WT |
| 3 | 71/F | W | I | 1.57 | 1.22 | 0.74 | GAT | 21 | 60/M | M | IV | 1.24 | 0.79 | 0.53 | WT |
| 4 | 54/F | W | I | 1.07 | 0.57 | 0.58 | WT | 22 | 41/F | M | II | 1.27 | 0.36 | 0.55 | WT |
| 5 | 66/M | W | III | 1.78 | 1.13 | 1.10 | GAT | 23 | 60/F | M | IV | 0.47 | 0.72 | 1.02 | GAT |
| 6 | 75/M | M | III | 1.72 | 0.36 | 0.81 | GTT | 24 | 74/M | M | III | 1.11 | 0.72 | 0.64 | GTT |
| 7 | 63/F | M | IV | 1.09 | 0.50 | 0.62 | CGT | 25 | 71/F | M | III | 1.39 | 0.72 | 0.67 | GTT |
| 8 | 63/M | M | I | 1.25 | 0.67 | 0.49 | GTT | 26 | 61/M | M | IV | 0.95 | 0.19 | 0.67 | GAT |
| 9 | 73/M | M | IV | 1.72 | 0.74 | 1.00 | GAT | 27 | 73/M | P | III | 1.17 | 0.58 | 0.62 | GAT |
| 10 | 79/M | M | III | 1.15 | 0.72 | 0.57 | GAT | 28 | 66/M | P | IV | 0.99 | 0.68 | 0.26 | WT |
| 11 | 73/F | M | I | 1.86 | 1.44 | 0.52 | GAT | 29 | 72/M | Meta | IV | 0.92 | 0.62 | 1.07 | GTT |
| 12 | 55/M | M | IV | 1.05 | 0.32 | 0.38 | GTT | 30 | 80/M | Meta | IV | 2.21 | 2.45 | 0.89 | GAT |
| 13 | 52/M | M | III | 2.32 | 1.30 | 1.75 | WT | 31 | 72/M | Meta | IV | 1.58 | 0.83 | 1.06 | GTT |
| 14 | 55/F | M | III | 1.48 | 0.23 | 0.60 | GAT | 32 | 69/M | Meta | IV | 1.11 | 0.36 | 0.87 | GAT |
| 15 | 63/F | M | IV | 1.1 | 0.58 | 0.80 | CGT | 33 | 46/F | Meta | IV | 1.48 | 1.12 | 1.22 | GTT |
| 16 | 57/M | M | IV | 0.69 | 0.26 | 0.60 | GAT | | | | | | | | |
| 17 | 69/F | M | III | 1.56 | 1.03 | 1.25 | GAT | | | | | | | | |
| 18 | 54/M | M | III | 1.41 | 0.47 | 0.99 | CGT | | | | | | | | |

[a]Histology: W, well; M, moderately; P, poorly differentiated tubular adenocarcinoma; Meta, metastatic adenocarcinoma; [b]stage according to the classification of WHO; [c] all the K-*ras* mutations involved codon 12. WT, wild type.

**Figure 2** Relationship between the expression levels of the *rhoC* gene in ductal adenocarcinoma of the pancreas and clinicopathological findings. (**A**) Expressions in tumour tissues (T) and in non-tumorous portions (N); (**B**) expressions in primary tumours (P) and in metastatic lesions (M); (**C**) tumours with perineural invasion and those without perineural invasion; (**D**) tumours with lymph node metastasis and without metastasis. *$P < 0.001$; **$P < 0.05$

0.82 ± 0.36 and 0.49 ± 0.20 respectively) (Figure 2A). There were no significant differences in the expression levels of the *rho*A or B genes between carcinoma tissues and non-malignant portions (data not shown). Metastatic lesions showed significantly higher *rho*C mRNA levels (1.02 ± 0.14) than primary pancreatic carcinoma tissues (0.78 ± 0.38, Figure 2B). These levels were not different between liver metastasis and peritoneal dissemination. The expression of the *rho*C gene in tumours with perineural invasion was 1.04 ± 0.34 and that in primary tumours with lymph node metastasis exhibited 0.86 ± 0.38. These expression levels were significantly higher than those without perineural invasion (0.61 ± 0.18) and those without metastasis (0.57 ± 0.09) respectively (Figures 2c and 2d).

**Figure 3** Correlation among the expressions of the *rho*A, B and C genes

There were no significant associations between *rho* gene expressions and other clinicopathological findings such as tumour size, location, age or sex (data not shown). Correlations between *rho* A, B and C expressions are shown in Figure 3. Gene expressions between *rho*A and *rho*B showed moderately positive correlation (correlation coefficient $r = 0.68$; $P < 0.0001$). Weakly positive correlation was observed between *rho*B and *rho*C expressions ($r = 0.50$; $P < 0.005$), and between *rho*A and *rho*C expressions ($r = 0.50$; $P < 0.005$).

**Figure 4** Patient survival in relation to *rho*C expression. The patients were divided into two groups by *rho*C expression at 0.82, which corresponded to the mean value in carcinoma tissues. —, *rho*C < 0.82 ($n = 17$); ·····, *rho*C ≥ 0.82 ($n = 14$). $P < 0.05$

**Figure 5** Histology in relation to *rho*C expression. In spite of being a well-differentiated subtype, Case 5, which is one of the high-expression group (1.10), showed perineural invasion (**A**). Case 28 is a poorly differentiated adenocarcinoma in the low-expression group (0.26). Perineural invasion was not observed (**B**). H & E staining. A, × 200; B, × 100

© Cancer Research Campaign 1998

As the mean value of *rho*C expression in carcinoma tissues was 0.82, cases were divided into two groups at this level – high expression and low expression. Patients in the high expression group revealed significantly poorer prognosis than the patients in the low expression group ($P < 0.05$, Fig. 4). Although case 5 was a well-differentiated adenocarcinoma, it was one of the high-expression group with perineural invasion (Figure 5a) and lymph node metastasis (data not shown) and revealed poor prognosis (survival period was 186 days). In contrast, case 28 was a poorly differentiated carcinoma but was one of the low-expression group without perineural invasion (Figure 5b) and the patient survived for 462 days after surgical resection. No mutation was found in the fragments of the *rho*A, B or C genes examined either by PCR-SSCP or by direct sequencing (data not shown).

### K-*ras* gene mutation

Mutation of the K-*ras* gene was found in 27 out of 33 (81.8%) pancreatic carcinoma tissues both by PCR-SSCP and by direct sequencing. There were no mutations in any of the non-tumorous portions examined. Sequence analysis revealed that all the K-*ras* mutations detected were at codon 12. Mutational patterns were from GGT to GAT in 13, GTT in ten, and CGT in four cases (Table 2). The expression levels of the *rho*C gene in tumour tissues with K-*ras* mutation were not significantly different from those without ($0.81 \pm 0.55$ and $0.83 \pm 0.32$ respectively). The presence of K-*ras* gene mutation had no relationship with clinicopathological findings (data not shown).

### DISCUSSION

In the present study we examined the expression levels of the *rho*A, B and C genes, and mutation status of the K-*ras* gene and the three *rho* gene isoforms in human ductal adenocarcinoma of the pancreas. Although the *rho* genes have been suspected to be involved in cell transformation, there are few reports about *rho* gene expressions in human tumour tissues. Recently, in vitro assay revealed that activated RhoA protein is necessary for the motility of keratinocytes induced by hepatocyte growth factor (Takaishi et al, 1994) and that the invasive activity of rat MM1 cells across the mesothelial cell monolayer is inhibited by *Clostridium boturinum* exo-enzyme C3 that specifically inactivates Rho proteins (Imamura et al, 1996). Serum-dependent invasional activity of hepatoma cells is regulated by activated RhoA protein (Yoshioka et al, 1995). In the present study, *rho*C gene expression was significantly higher in tumour portions than in non-tumour portions of the pancreas. Furthermore, tumours with lymph node metastasis and those with perineural invasion exhibited significantly higher expression of the *rho*C gene than those without these manifestations, irrespective of histological grading or differentiation. Levels of the *rho*C mRNA were also significantly higher in metastatic lesions than in primary pancreatic carcinomas. These suggest that overexpression of the *rho*C gene occurs during pancreatic carcinogenesis and that its overexpression may be associated with the invasive characteristics of pancreatic cancer. As matched pairs of primary and metastatic lesions could not be compared in the present study, it remains to be elucidated whether overexpression of the *rho*C gene is directly associated with metastatic process or not. In the current study, *rho* gene expressions were only moderately associated each other. RhoA and RhoC proteins are predominantly associated with the submembranous actin network and RhoB is found in association with multivesicular bodies (Robertson et al, 1995). Although *rho*A and *rho*C expression levels are not different in human breast cancer cell lines and normal mammary epithelial cells, *rho*B expressions show a dramatic variation and are implicated in cell proliferation (de Cremoux et al, 1994). RhoA has weakly transforming activity in NIH3T3 cells (Avraham and Weinberg, 1989). The *rho*B gene is an immediate–early response gene for epidermal growth factor and the v-*src* oncogene (Jahner and Hunter, 1991). Recently, it has been suggested that RhoC regulates microfilament organization in the apical pole of intestinal epithelial cells (Nusrat et al, 1995). Target proteins for each Rho protein could be different (Watanabe et al, 1996) and the function in carcinogenesis, if any, may be different in individual Rho proteins.

The incidence of K-*ras* gene mutation in ductal adenocarcinoma of the pancreas in the present study is similar to other previous reports (Almoguera et al, 1988; Smit et al, 1988; Hruban et al, 1993). It is reported that oncogenic Ras may cause some aspects of the malignant phenotype by deregulating the Rho family protein function (Khosravi-Far and Der, 1994). In the present study, the expression levels of the *rho* genes have not been affected by the mutational status of the K-*ras* gene. The activities of Ras and Rho family proteins may be coordinately regulated by dual-function proteins such as mCDC25 and p190 through GTP/GDP cycles (Khosravi-Far and Der, 1994). Thus, it remains possible that the Ras mutation status influences the Rho function by upregulating the level of the active GTP-bound form irrespective of Rho protein amount. In the current study mutational change was not detected in any of the *rho* genes. Mutational activation of the *rho*A gene is not observed in lung, breast, colon and ovarian tumours (Moscow et al, 1994). Thus, *rho* genes are unlikely to be activated by mutation during pancreatic carcinogenesis. However, sequencing of entire coding regions have to be determined to confirm whether mutation exists or not in the *rho* genes. In conclusion, the expression level of the *rho* genes in ductal adenocarcinoma of the pancreas is not affected by K-*ras* gene mutation and *rho*C gene overexpression may play a role in tumour invasion resulting in poorer prognosis.

### ACKNOWLEDGEMENT

This work was supported by Grants-in-Aid from the Ministry of Education, Science and Culture of Japan to MF.

### ABBREVIATIONS

PCR, polymerase chain reaction; RT-PCR, PCR after reverse transcription; SSCP, single-strand conformation polymorphism

#### REFERENCES

Almoguera C, Shibata D, Forrester K, Martin J and Perucho M (1988) Most human carcinoma of the exocrine pancreas contain mutant c-K-ras genes. *Cell* **53**: 549–554

Arao S, Suwa H, Mandai M, Tashiro H, Miyazaki K, Okamura H, Nomura H, Hiai H and Fukumoto M (1994) Expression of multidrug resistance gene and localization of P-glycoprotein in human primary ovarian cancer. *Cancer Res* **54**: 1355–1359

Avraham H and Weinberg RA (1989) Characterization and expression of the human rhoH12 gene product. *Mol Cell Biol* **9**: 2058–2066

Bar-Sagi D and Feramisco JR (1986) Induction of membrane ruffling and fluid-phase pinocytosis in quiscent fibroblasts by ras proteins. *Science* **233**: 1061–1068

© Cancer Research Campaign 1998 

Barton CM, Staddon SL, Hughes CM, Hall PA, O'Sullivan C, Kloppel G, Theise B, Russel RCG, Neoptolemos J, Williamson RCN, Lane DP and Lemoine NR (1991) Abnormalities of the p53 tumor suppressor gene in human pancreatic cancer. *Br J Cancer* **64**: 1076–1082

Boguski MS and McCormick F (1993) Proteins regulating ras and its relatives. *Nature* **366**: 643–654

Chardin P, Madaule P and Tavitian A (1988) Coding sequence of human rho cDNAs clone 6 and clone 9. *Nucleic Acids Res* **16**: 2717

De Cremoux P, Gauville C, Closson V, Linares G, Calvo F, Tavitian A and Olofsson B (1994) EGF modulation of the ras-related *rho*B gene expression in human breast-cancer cell lines. *Int J Cancer* **59**: 408–415

Gibson JB and Sobin LH (1978) Histological typing of tumours of the liver, biliary tract and pancreas. *WHO, International Histological Classification of Tumours, no. 20*, World Health Organization: Geneva.

Hall A (1990) The cellular functions of small GTP-binding proteins. *Science* **249**: 635–640

Hohne MW, Halatch ME, Kahl GF and Weinel RJ (1992) Frequent loss of expression of the potential tumor suppressor gene DCC in ductal pancreatic adenocarcinoma. *Cancer Res* **52**: 2616–2619

Hruban RH, Van Mansfeld ADM, Offerhaus GJA, Van Weering DHJ, Allison DC, Goodman SN, Kensler DW, Bose KK, Cameron J and Bos JL (1993) K-ras oncogene activation in adenocarcinoma of the human pancreas. *Am J Pathol* **143**: 545–554

Imamura F, Shinkai K, Mukai M, Yoshioka K, Komagome R, Iwasaki T and Akedo H (1996) rho-mediated protein tyrosine phosphorylation in lysophosphatidic-acid-induced tumor-cell invasion. *Int J Cancer* **65**: 627–632

Jahner D and Hunter T (1991) The ras-related gene rhoB is an immediate-early gene inducible by v-Fps, epidermal growth factor, and platelet-derived growth factor in rat fibroblasts. *Mol Cell Biol* **11**: 3682–3690

Khosravi-Far R and Der CJ (1994) The ras signal transduction pathway. *Cancer Meta Rev* **13**: 67–89

Moscow JA, He R, Gnarra JR, Knutsen T, Weng Y, Zhao WP, Whang-Peng J, Linehan WM and Cowan KH (1994) Examination of human tumours for rho A mutations. *Oncogene* **9**: 189–194

Nusrat A, Giry M, Turner JR, Colgan SP, Parkos CA, Carnes D, Lemichez E, Boquet P and Madara JL (1995) Rho protein regulates tight junctions and perijunctional actin organization in polarized epithelia. *Proc Natl Acad Sci USA* **921**: 10629–10633

Olson MF (1996) Guanine nucleotide exchange factors for the Rho GTPases: a role in human disease? *J Mol Med* **74**: 563–571

Orita M, Iwahana H, Kanazawa H, Hayashi K and Sekiya T (1989) Detection of polymorphisms of human DNA by gel electrophoresis as single-strand coformation polymorphisms. *Proc Natl Acad Sci USA* **86**: 2766–2770

Prendergast GC, Khosravi-Far R, Solski PA, Kurzawa H, Lebowitz PF and Der CJ (1995) Critical role of Rho in cell transformation by oncogenic Ras. *Oncogene* **10**: 2289–2296

Ridley AJ and Hall A (1992) The small GTP-binding protein rho regulates the assembly of focal adhesions and actin stress fibers in response to growth factors. *Cell* **70**: 389–399

Robertson D, Paterson HF, Adamson P, Hall A and Monaghan P (1995) Ultrastructural localization of ras-related proteins using epitope-tagged plasmids. *J Histochem Cytochem* **43**: 471–480

Scarpa A, Cappeli P, Mukai K, Zamboni G, Oda T, Lacono C and Hirohashi S (1993) Pancreatic carcinoma frequently show p53 gene mutations. *Am J Pathol* **142**: 1534–1543

Seymour AB, Hruban RH, Redston M, Caldas C, Powell SM, Kinzler KW, Yeo CJ and Kern SE (1995) Allelotype of pancreatic adenocarcinoma. *Cancer Res* **54**: 2761–2764

Smit VT, Boot AJ, Smits AA, Fleuren GJ, Cornellisse CJ and Bos JL (1988) K-ras codon 12 mutations occur very frequently in pancreatic adenocarcinoma. *Nucleic Acids Res* **16**: 7773–7782

Suwa H, Yoshimura T, Yamaguchi N, Kanehira K, Manabe T, Imamura M, Hiai H and Fukumoto M (1994) K-ras and p53 alterations in genomic DNA and transcripts of human pancreatic adenocarcinoma cell lines. *Jpn J Cancer Res* **85**: 1005–1014

Takaishi K, Sasaki T, Kato M, Yamochi W, Kuroda S, Nakamura T, Takeichi M and Takai Y (1994) Involvement of Rho p21 small GTP-binding protein and its regulator in the HGF-induced cell motility. *Oncogene* **9**: 273–279

Warshaw AL and Casttilo CF (1992) Pancreatic carcinoma. *N Engl J Med* **326**: 455–465

Watanabe G, Saito Y, Madaule P, Ishizaki T, Fujisawa K, Morii N, Mukai H, Ono Y, Kakizuka A and Narumiya S (1996) Protein kinase N (PKN) and PKN-related protein Rhophilin as targets of small GTPase Rho. *Science* **271**: 645–648

Yeramian P, Chardin P, Madaule P and Tavitian A (1987) Nucleotide sequence of human rho cDNA clone 12. *Nucleic Acids Res* **15**: 1869

Yoshioka K, Imamura F, Shinkai K, Miyoshi J, Ogawa H, Mukai M, Komagome R and Akedo H (1995) Participation of rho p21 in serum-dependent invasion by rat ascites hepatoma cells. *FEBS Lett* **372**: 25–28

© Cancer Research Campaign 1998